# Relationship between plasma high mobility group box-1 protein levels and clinical outcomes of aneurysmal subarachnoid hemorrhage

**DOI:** 10.1186/1742-2094-9-194

**Published:** 2012-08-11

**Authors:** Xiang-Dong Zhu, Jing-Sen Chen, Feng Zhou, Qi-Chang Liu, Gao Chen, Jian-Min Zhang

**Affiliations:** 1 Department of Neurosurgery, The Second Affiliated Hospital, School of Medicine, Zhejiang University, 88 Jiefang Road, Hangzhou, 310000, PR China

**Keywords:** Aneurysmal subarachnoid hemorrhage, Cerebrovasospasm, Functional outcome, High-mobility group box 1, Mortality

## Abstract

**Background:**

High-mobility group box 1 (HMGB1), originally described as a nuclear protein that binds to and modifies DNA, is now regarded as a central mediator of inflammation by acting as a cytokine. However, the association of HMGB1 in the peripheral blood with disease outcome and cerebrovasospasm has not been examined in patients with aneurysmal subarachnoid hemorrhage.

**Methods:**

In this study, 303 consecutive patients were included. Upon admission, plasma HMGB1 levels were measured by ELISA. The end points were mortality after 1 year, in-hospital mortality, cerebrovasospasm and poor functional outcome (Glasgow Outcome Scale score of 1 to 3) after 1 year.

**Results:**

Upon admission, the plasma HMGB1 level in patients was statistically significantly higher than that in healthy controls. A multivariate analysis showed that the plasma HMGB1 level was an independent predictor of poor functional outcome and mortality after 1 year, in-hospital mortality and cerebrovasospasm. A receiver operating characteristic curve showed that plasma HMGB1 level on admission statistically significantly predicted poor functional outcome and mortality after 1 year, in-hospital mortality and cerebrovasospasm of patients. The area under the curve of the HMGB1 concentration was similar to those of World Federation of Neurological Surgeons (WFNS) score and modified Fisher score for the prediction of poor functional outcome and mortality after 1 year, and in-hospital mortality, but not for the prediction of cerebrovasospasm. In a combined logistic-regression model, HMGB1 improved the area under the curve of WFNS score and modified Fisher score for the prediction of poor functional outcome after 1 year, but not for the prediction of mortality after 1 year, in-hospital mortality, or cerebrovasospasm.

**Conclusions:**

HMGB1 level is a useful, complementary tool to predict functional outcome and mortality after aneurysmal subarachnoid hemorrhage. However, HMGB1 determination does not add to the accuracy of prediction of the clinical outcomes.

## Background

Subarachnoid hemorrhage (SAH) following cerebral aneurysm rupture is associated with high rates of morbidity and mortality [1]. Early prognostication of the risk of death or of a poor long-term outcome would enable optimized care and improved allocation of health-care resources. Several scales of outcome prediction, including the World Federation of Neurological Surgeons (WFNS) score [2] and Fisher score [3], are known to be associated with poor clinical outcomes. However, a readily measurable predictive marker predicting clinical outcomes in patients with SAH would be helpful for early prognostication and risk stratification, and is attracting increasing attention as a potential predictor of outcome in SAH [4].

High-mobility group box 1 (HMGB1) is constitutively expressed in the nuclei of eukaryotic cells. It belongs to a family of high mobility group nuclear proteins that were described in the 1970s as gene regulators that bind to and change the configuration of DNA [5,6]. It later became evident that HMGB1 is actively secreted from cells, has cytokine activities and is a late mediator of endotoxin lethality in mice [7]. Passive release of HMGB1 from necrotic cells also triggers inflammation [8]. Receptors for HMGB1 signaling include receptors for advanced glycation end-products and Toll-like receptors. HMGB1 is a central actor in the inflammatory network because it is induced by a number of cytokines and itself induces a series of inflammatory reactions [9-12].

HMGB1 is widely expressed in various tissues, including the brain [13-15]. The local extracellular accumulation of this cytokine in the brain, as a result of its intracerebroventricular administration or its release from dying neurons during cerebral ischemia, elicits local inflammatory events in mice [16-18]. Moreover, HMGB1 also promotes neuroinflammation in postischemic rat brain [17] and in the neurodegenerative processes associated with Alzheimer’s disease [19,20]. Taken together, HMGB1 behaves as a typical immunoregulatory cytokine by promoting astrocyte activation and thus the production of a potent mixture of bioactive protein factors involved in inflammatory/immune responses in the brain [21].

HMGB1 is increased in the cerebrospinal fluid from meningitis patients [22] and in the serum of cerebral ischemia patients [23]. Recent data have identified HMGB1 in the cerebrospinal fluid as a potential biomarker of neurological outcome following SAH in humans [24,25], suggesting HMGB1 may represent a marker of neurological injury. However, no published information exists to date about the association between HMGB1 in the peripheral blood and disease outcome and cerebrovasospasm after SAH. The present study aimed to investigate the ability of plasma HMGB1 to predict the disease outcome and cerebrovasospasm in patients with aneurysmal SAH.

### Subjects and methods

#### Study population

Between July 2008 and March 2010, all patients with aneurysmal SAH confirmed by computerized tomography (CT) angiography with or without digital subtraction angiography who were admitted to the Department of Neurosurgery, Second Affiliated Hospital, School of Medicine, Zhejiang University were evaluated in the study. Inclusion criteria were clinical history of SAH within the last 24 hours before admission and the treatment by surgery or coiling within the 48 hours after admission. Exclusion criteria were age less than 18 years, existing previous head trauma, neurological diseases including ischemic or hemorrhagic stroke, use of antiplatelet or anticoagulant medication, and presence of other prior systemic diseases including uremia, liver cirrhosis, malignancy, chronic heart or lung disease, diabetes mellitus and hypertension.

A control group consisted of 150 healthy subjects without existing previous head trauma, neurological diseases including ischemic or hemorrhagic stroke, use of antiplatelet or anticoagulant medication, and presence of other prior systemic diseases including uremia, liver cirrhosis, malignancy, chronic heart or lung disease, diabetes mellitus and hypertension.

Written informed consent to participate in the study was obtained from the subjects or their relatives. This protocol was approved by the Ethics Committee of The Second Affiliated Hospital, School of Medicine, Zhejiang University before implementation.

#### Clinical and radiological assessment

On arrival at the emergency department, a detailed history of vascular risk factors, concomitant medication, Glasgow Coma Scale (GCS) score, body temperature, heart rate, respiratory rate and blood pressure were taken. At admission, clinical severity was assessed using WFNS score [2]. The initial CT was classified according to the modified Fisher score [3]. All CT scans were performed according to the neuroradiology department protocol. Investigators who read them were blinded to clinical information.

#### Patient management

The type of treatment (surgery or coiling) was decided according to both location and size of the aneurysm by the neurosurgeon and the neuroradiologist. All patients received intravenous nimodipine at a dose of 2 mg/hour from admission until at least day 14, except during periods of uncontrolled increased intracranial pressure during which intravenous nimodipine was discontinued. Seizures were systematically prevented by sodium valproate (200 mg × 3, per os). After surgery or coiling, those patients who had delayed ischemic neurological deficit or cerebrovasospasm were managed with 'triple H' therapy (hypertension with a mean arterial pressure goal greater than 100 mmHg, hypervolemia and hemodilution with a goal hematocrit of 30) through 12 days after hemorrhage. An external ventricular drain was inserted in the case of hydrocephalus on CT and in patients with a high WFNS grade (WFNS score of 3 to 5). Increased intracranial pressure was treated by cerebrospinal fluid drainage, mechanical ventilation, reinforcement of sedation, and, rarely, moderate hypothermia. CT was performed whenever clinical deterioration occurred to search for secondary complications such as hydrocephalus or ischemia.

Clinical onset of cerebral vasospasm was defined as the acute onset of a focal neurologic deficit or a change in the GCS score of 2 or more points. All suspected cases of cerebral vasospasms were confirmed by CT angiography and were then taken to the interventional radiology suite for cerebral angiography. Each vasospasm episode was treated with intra-arterial administration of nimodipine as recently described. This therapy was repeated if necessary. Balloon angioplasty was used as a second-line therapy when nimodipine was judged insufficient. Computed tomography ischemia was referred to as delayed ischemia attributed to vasospasm.

#### Determination of HMGB1 in plasma

Informed consents were obtained from the study population or family members in all cases before blood was collected. In the control group, venous blood was drawn at study entry. In the SAH patients, venous blood was drawn on admission. The blood samples were immediately placed into sterile EDTA test tubes and centrifuged at 1500 g for 20 minutes at 4°C to collect plasma. Plasma was stored at −70°C until assayed. The concentration of HMGB1 in plasma was analyzed by ELISA using commercial kits (SHINO-TEST Corporations, Kanagawa, Japan) in accordance with the manufacturers’ instructions. The blood samples were run in duplicate. Researchers running ELISAs were blinded to all patient details.

#### End point

Participants were followed up until death or completion of 1 year after SAH. Their primary outcome was death (at 1 year or in-hospital) and their secondary outcomes were vasospasm and functional outcome at 1 year. The functional outcome was defined by Glasgow outcome scale (GOS) score. GOS was defined as follows: 1 = death; 2 = persistent vegetative state; 3 = severe disability; 4 = moderate disability; and 5 = good recovery [26]. GOS Scores were dichotomized in good and poor functional outcomes (GOS of 4 to 5 vs. GOS of 1 to 3). For follow-up, we used structure telephone interviews performed by one doctor, blinded to clinical information, and copeptin levels.

#### Statistical analysis

Statistical analysis was performed with SPSS 10.0 (SPSS Inc., Chicago, IL, USA) and MedCalc 9.6.4.0. (MedCalc Software, Mariakerke, Belgium). The normality of data distribution was assessed by the Kolmogorovor-Smirnov test or Shapiro-Wilk test. All values are expressed as mean ± standard deviation or counts (percentage) unless otherwise specified. Comparisons were made by using (1) chi-square test or Fisher exact test for categorical data, (2) unpaired Student *t* test for continuous normally distributed variables, and (3) the Mann–Whitney U-test for continuous non-normally distributed variables. The correlations of HMGB1 with WFNS grade and Fisher grade were assessed by Spearman’s correlation coefficient. The relations of HMGB1 to the poor functional outcome (GOS 1 to 3), death and cerebrovasospasm were assessed in a binary logistic-regression model. For multivariate analysis, we included the significantly different outcome predictors as assessed in univariate analysis. A receiver operating characteristic curve was configured to establish the cutoff point of plasma HMGB1 with the optimal sensitivity and specificity for predicting the poor functional outcome (GOS 1 to 3), death and cerebrovasospasm. In a combined logistic-regression model, we estimated the additive benefit of HMGB1 to other predictors (WFNS grade and Fisher grade). A *P* value of less than 0.05 was considered statistically significant.

## Results

### Study population characteristics

**Figure 1:**
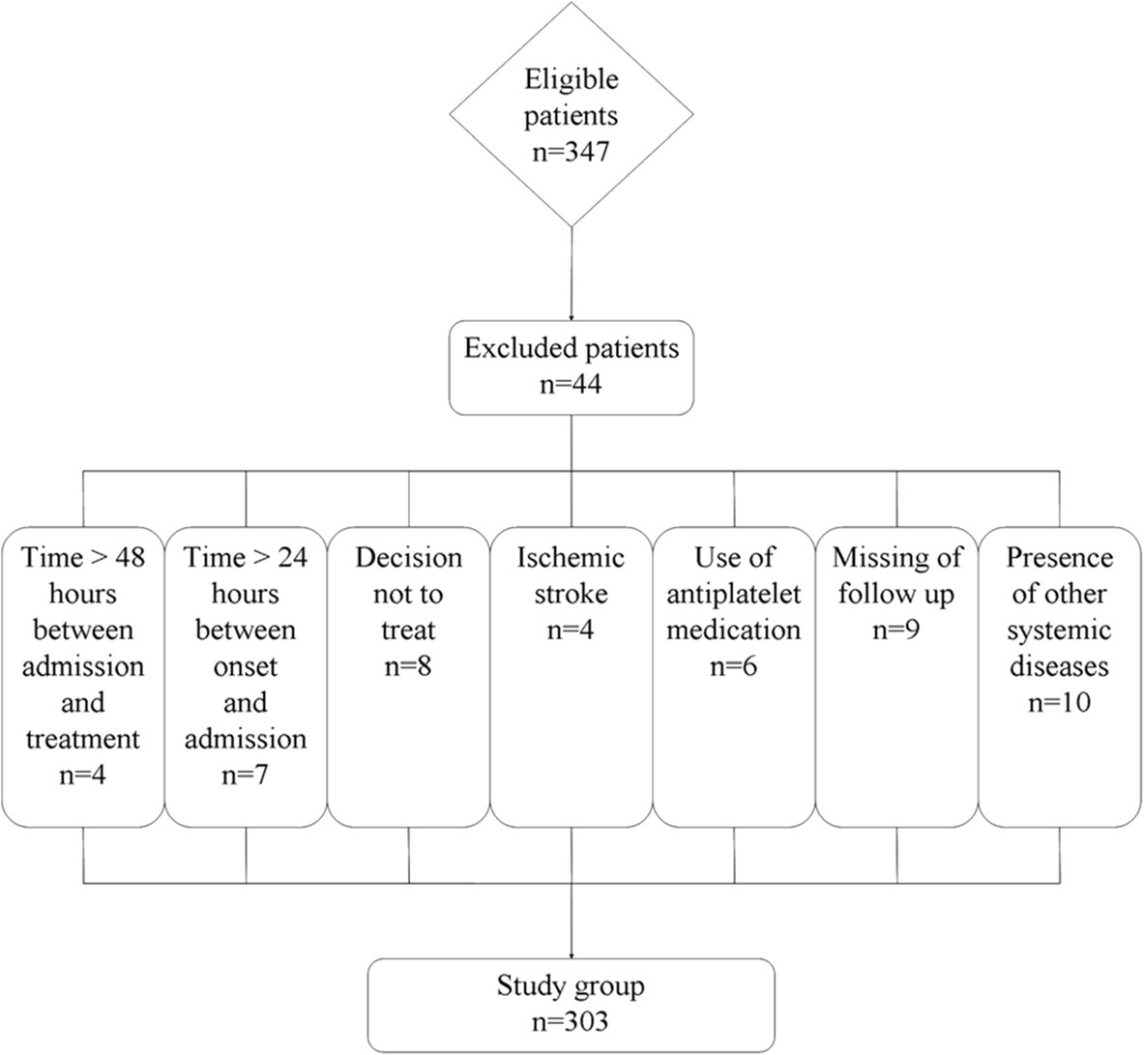
Graph documenting patients’ entry into the study from screening.

During the recruitment period 347 patients were admitted with an initial diagnosis of aneurysmal SAH, 312 (89.9%) patients fulfilled the inclusion criteria, and adequate data on admission and follow-up were available for 303 individuals (87.3%) who were finally included in the analysis (Figure 1). Table 1 summarizes the demographic, clinical, laboratory and radiological data of the patients. One hundred and fifty healthy subjects were eligible as controls. The intergroup differences in the age and sex were not statistically significant. After SAH, plasma HMGB1 level on admission in patients was statistically significantly higher than that in healthy controls (8.5 ± 3.6 ng/mL vs. 1.3 ±0.4 ng/mL; *P* < 0.001). Moreover, a significant correlation emerged between plasma HMGB1 level and WFNS score (r = 0.635, *P* < 0.001), as well as between plasma HMGB1 level and modified Fisher score (r = 0.624, *P* < 0.001).

**Table 1:** The characteristics for 303 patients

**Characteristic**	
Sex (male/female)	131/172
Age (years)	43.9 ± 12.4
World Federation of Neurological Surgeons score on admission	2.3 ± 1.2
Modified Fisher score on admission	2.7 ± 1.0
Aneurysmal location	
Posterior communication artery	83 (27.4%)
Internal carotid artery	43 (14.2%)
Anterior communication artery	66 (21.8%)
Middle cerebral artery	45 (14.9%)
Anterior cerebral artery	35 (11.6%)
Posterior cerebral artery	23 (7.6%)
Vertebral artery	8 (2.6%)
Surgery	186 (61.4%)
Aneurysmal size (mm)	7.2 ± 4.9
Rebleeding	16 (5.3%)
Acute hydrocephalus	90 (29.7%)
Intracerebral hemorrhage	39 (12.9%)
Intraventricular hemorrhage	72 (23.8%)
External ventricular drain	109 (36.0%)
Angiographic vasospasm	131 (43.2%)
Computed tomography ischemia	50 (16.5%)
Admission time (hours)	4.7 ± 3.6
Plasma-sampling time (hours)	6.7 ± 4.4
Seizure	44 (14.5%)
Plasma C-reactive protein level (mg/L)	7.1 ± 2.7
plasma D-dimer level (mg/L)	2.1 ± 0.9
Plasma HMGB1 level (ng/mL)	8.5 ± 3.6

Numerical variables were presented as mean ± standard deviation. Categorical variables were expressed as counts (percentage). HMGB1, high mobility group box-1.

### One-year mortality prediction

Forty-two patients (13.9%) died from SAH in 1 year. Higher plasma HMGB1 level was associated with 1-year mortality, as well as other variables shown in Table 2. When the above variables that were found to be significant in the univariate analysis were introduced into the logistic model, a multivariate analysis selected WFNS score (odds ratio, 7.491; 95% confidence interval (CI) 1.361 to 21.351; *P* = 0.001), modified Fisher score (odds ratio, 9.292; 95% CI 2.346 to 23.318; *P* = 0.005) and plasma HMGB1 level (odds ratio, 2.117; 95% CI 1.109 to 7.230; *P* = 0.002) as the independent predictors for 1-year mortality of patients.

**Table 2:** The factors associated with 1-year mortality

	**Non-survivors**	**Survivors**	***P*value**
	**(n = 42)**	**(n = 261)**	
Sex (male/female)	18/24	113/148	0.958
Age (years)	45.4 ± 13.4	43.6 ± 12.2	0.389
WFNS score on admission	4.0 ± 0.7	2.1 ± 1.0	<0.001
Modified Fisher score on admission	4.2 ± 0.6	2.5 ± 0.8	<0.001
Aneurysmal location			0.614
Posterior communication artery	8 (19.0%)	75 (28.7%)	
Internal carotid artery	6 (14.3%)	37 (14.2%)	
Anterior communication artery	9 (21.4%)	57 (21.8%)	
Middle cerebral artery	7 (16.7%)	38 (14.6%)	
Anterior cerebral artery	6 (14.3%)	29 (11.1%)	
Posterior cerebral artery	4 (9.5%)	19 (7.3%)	
Vertebral artery	2 (4.8%)	6 (2.3%)	
Surgery	21 (50.0%)	165 (63.2%)	0.102
Aneurysmal size (mm)	11.1 ± 5.3	6.6 ± 4.5	<0.001
Rebleeding	10 (23.8%)	6 (2.3%)	<0.001
Acute hydrocephalus	25 (59.5%)	65 (24.9%)	0.001
Intracerebral hemorrhage	19 (45.2%)	20 (7.7%)	<0.001
Intraventricular hemorrhage	37 (88.1%)	35 (13.4%)	<0.001
External ventricular drain	38 (90.5%)	71 (27.2%)	<0.001
Angiographic vasospasm	38 (90.5%)	93 (35.6%)	<0.001
Computed tomography ischemia	18 (42.9%)	32 (12.3%)	<0.001
Admission time (hours)	5.5 ± 4.4	4.6 ± 3.5	0.128
Seizure	9 (21.4%)	35 (13.4%)	0.171
Plasma C-reactive protein level (mg/L)	8.7 ± 3.3	6.9 ± 2.6	<0.001
plasma D-dimer level (mg/L)	2.4 ± 1.0	2.0 ± 1.0	0.015
Plasma HMGB1 level (ng/mL)	12.7 ± 3.2	7.9 ± 3.2	<0.001

Numerical variables are presented as mean ± standard deviation. Categorical variables are expressed as counts (percentage). Numerical variables were analyzed by Mann–Whitney U-test or unpaired Student *t* test. Categorical variables were analyzed by chi-square test or Fisher exact test. HMGB1, high mobility group box-1; n, number of patients; WFNS, World Federation of Neurological Surgeons.

**Figure 2:**
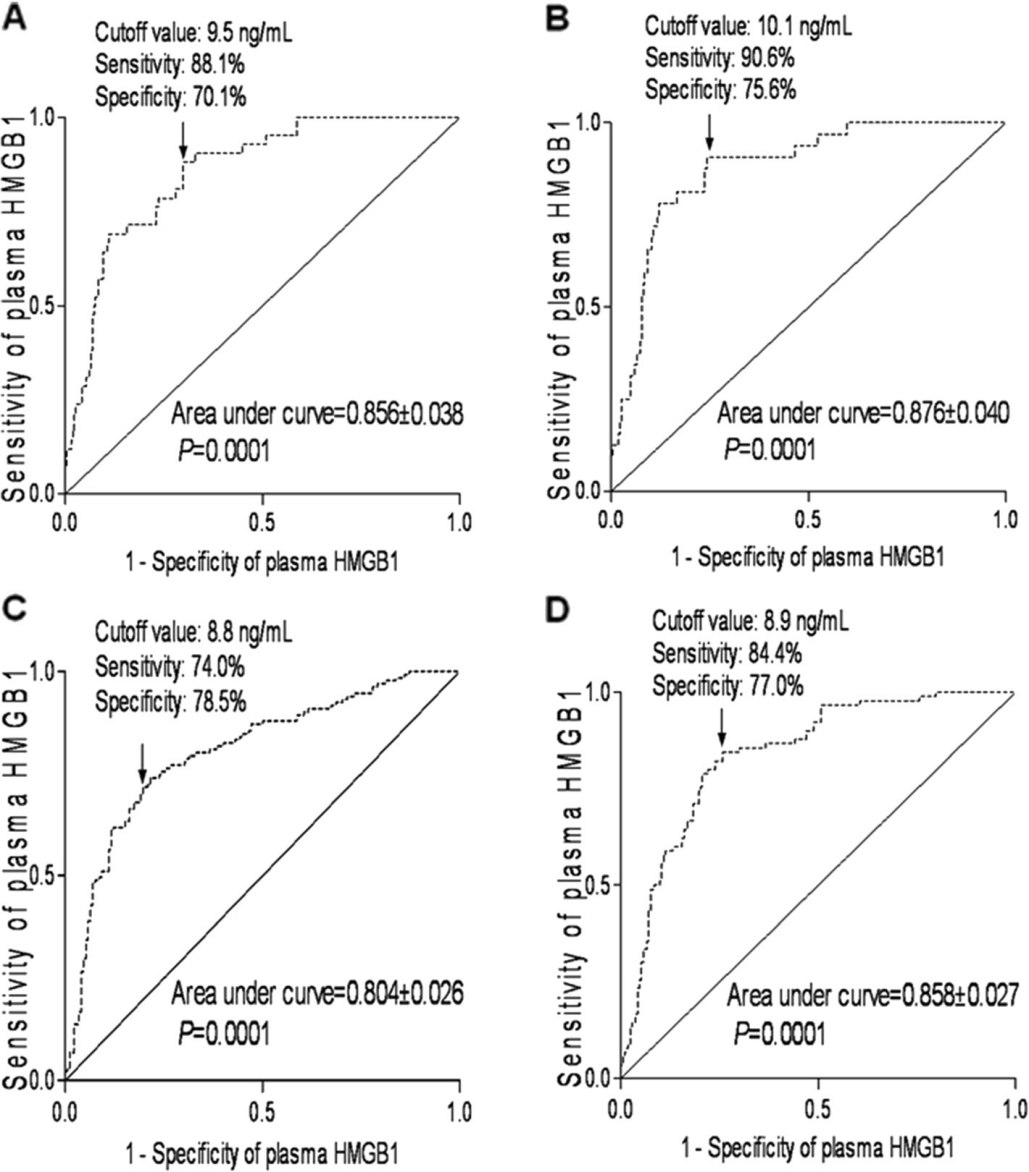
Graph showing receiver operating characteristic curve analysis of plasma high mobility group box-1 (HMGB1) level for (A) 1-year mortality, (B) in-hospital mortality, (C) cerebrovasospasm and (D) 1-year poor functional outcome.

A receiver operating characteristic curve showed that plasma HMGB1 level on admission statistically significantly predicted 1-year mortality of patients (Figure 2A). The predictive value of the HMGB1 concentration was similar to those of WFNS score and modified Fisher score (Table 3). In a combined logistic-regression model, HMGB1 did not statistically significantly improve the area under the curve of WFNS score (*P* = 0.107) or modified Fisher score (*P* = 0.160).

**Table 3:** Receiver operating characteristic curve analysis of factors predicting the 1-year mortality among 303 patients

	**HMGB1**	**WFNS score**	**Modified Fisher score**
Criterion	>9.5 ng/mL	>3	>3
Area under curve	0.856	0.920	0.927
95% confidence interval	0.812 to 0.894	0.884 to 0.948	0.892 to 0.954
Sensitivity	88.1	78.6	88.1
95% confidence interval	74.4 to 96.0	63.2 to 89.7	74.4 to 96.0
Specificity	70.1	90.4	86.6
95% confidence interval	64.2 to 75.6	86.2 to 93.7	81.8 to 90.5
+ likelihood ratio	2.95	8.20	6.57
95% confidence interval	2.6 to 3.4	7.0 to 9.7	5.8 to 7.4
- likelihood ratio	0.17	0.24	0.14
95% confidence interval	0.07 to 0.4	0.1 to 0.5	0.06 to 0.3
*P* value	Reference	0.106	0.100

HMGB1, high mobility group box-1; WFNS, World Federation of Neurological Surgeons.

### In–hospital mortality prediction

Thirty-two patients (10.6%) died from SAH in the hospital. Higher plasma HMGB1 level was associated with in-hospital mortality, as well as other variables shown in Table 4. When the above variables that were found to be significant in the univariate analysis were introduced into the logistic model, a multivariate analysis selected WFNS score (odds ratio, 4.877; 95% CI 1.448 to 14.301; *P* = 0.002), modified Fisher score (odds ratio, 5.624; 95% CI 2.119 to 16.993; *P* = 0.008) and plasma HMGB1 level (odds ratio, 2.245; 95% CI 1.218 to 8.949; *P* = 0.005) as the independent predictors for in-hospital mortality of patients.

**Table 4:** The factors associated with in-hospital mortality

	**Non-survivors**	**Survivors**	***P*value**
	**(n = 32)**	**(n = 271)**	
Sex (male/female)	12/20	119/152	0.489
Age (years)	45.4 ± 12.9	43.7 ± 12.3	0.469
WFNS score on admission	3.9 ± 0.7	2.2 ± 1.1	<0.001
Modified Fisher score on admission	4.3 ± 0.6	2.6 ± 0.8	<0.001
Aneurysmal location			0.599
Posterior communication artery	5 (15.6%)	78 (28.8%)	
Internal carotid artery	6 (18.8%)	37 (13.7%)	
Anterior communication artery	8 (25.0%)	58 (21.4%)	
Middle cerebral artery	4 (12.5%)	41 (15.1%)	
Anterior cerebral artery	4 (12.5%)	31 (11.4%)	
Posterior cerebral artery	3 (9.4%)	20 (7.4%)	
Vertebral artery	2 (6.3%)	6 (2.2%)	
Surgery	17 (53.1%)	169 (62.4%)	0.310
Aneurysmal size (mm)	11.9 ± 5.1	6.7 ± 4.5	<0.001
Rebleeding	6 (18.8%)	10 (3.7%)	<0.001
Acute hydrocephalus	19 (59.4%)	71 (26.2%)	<0.001
Intracerebral hemorrhage	15 (46.9%)	24 (8.9%)	<0.001
Intraventricular hemorrhage	31 (96.9%)	41 (15.1%)	<0.001
External ventricular drain	30 (93.8%)	79 (29.2%)	<0.001
Angiographic vasospasm	30 (93.8%)	101 (37.3%)	<0.001
Computed tomography ischemia	16 (50.0%)	34 (12.6%)	<0.001
Admission time (hours)	5.4 ± 4.2	4.6 ± 3.6	0.276
Seizure	7 (21.9%)	37 (13.7%)	0.212
Plasma C-reactive protein level (mg/L)	9.0 ± 3.2	6.9 ± 2.6	<0.001
plasma D-dimer level (mg/L)	2.4 ± 1.1	2.0 ± 1.0	0.045
Plasma HMGB1 level (ng/mL)	13.1 ± 3.1	8.0 ± 3.2	<0.001

Numerical variables are presented as mean ± standard deviation. Categorical variables are expressed as counts (percentage). Numerical variables were analyzed by Mann–Whitney U-test or unpaired Student *t* test. Categorical variables were analyzed by chi-square test or Fisher exact test. HMGB1, high mobility group box-1; n, number of patients; WFNS, World Federation of Neurological Surgeons.

A receiver operating characteristic curve showed that plasma HMGB1 level on admission statistically significantly predicted in-hospital mortality of patients (Figure 2B). The predictive value of the HMGB1 concentration was similar to those of WFNS score and modified Fisher score (Table 5). In a combined logistic-regression model, HMGB1 did not statistically significantly improve the area under the curve of WFNS score (*P* = 0.140) or modified Fisher score (*P* = 0.161).

**Table 5:** Receiver operating characteristic curve analysis of factors predicting the in-hospital mortality among 303 patients

	**HMGB1**	**WFNS score**	**Modified Fisher score**
Criterion	>10.1 ng/mL	>3	>3
Area under curve	0.876	0.893	0.922
95% confidence interval	0.833 to 0.911	0.853 to 0.926	0.886 to 0.950
Sensitivity	90.6	75.0	90.6
95% confidence interval	75.0 to 97.9	56.6 to 88.5	75.0 to 97.9
Specificity	75.6	87.5	84.1
95% confidence interval	70.1 to 80.6	82.9 to 91.2	79.2 to 88.3
+ likelihood ratio	3.72	5.98	5.71
95% confidence interval	3.3 to 4.2	4.9 to 7.3	5.1 to 6.5
- likelihood ratio	0.12	0.29	0.11
95% confidence interval	0.04 to 0.4	0.1 to 0.6	0.04 to 0.3
*P* value	Reference	0.707	0.328

HMGB1, high mobility group box-1; WFNS, World Federation of Neurological Surgeons.

### Cerebrovasospasm prediction

One hundred and thirty-one (43.2%) suffered from cerebrovasospasm in the hospital. Higher plasma HMGB1 level was associated with cerebrovasospasm, as well as other variables shown in Table 6. When the above variables that were found to be significant in the univariate analysis were introduced into the logistic model, a multivariate analysis selected WFNS score (odds ratio, 3.890; 95% CI 1.230 to 8.421; *P* = 0.008), modified Fisher score (odds ratio, 4.713; 95% CI 1.689 to 15.106; *P* = 0.001) and plasma HMGB1 level (odds ratio, 1.249; 95% CI 1.132 to 1.871; *P* = 0.011) as the independent predictors for cerebrovasospasm of patients.

**Table 6:** The factors associated with cerebrovasospasm

	**Vasospasm**	**Non-vasospasm**	***P*value**
	**(n = 131)**	**(n = 172)**	
Sex (male/female)	61/70	70/102	0.307
Age (years)	43.8 ± 12.2	43.9 ± 12.5	0.973
WFNS score on admission	3.2 ± 1.1	1.7 ± 0.9	<0.001
Modified Fisher score on admission	3.5 ± 1.0	2.2 ± 0.6	<0.001
Aneurysmal location			0.813
Posterior communication artery	33 (25.2%)	50 (29.1%)	
Internal carotid artery	20 (15.3%)	23 (13.4%)	
Anterior communication artery	29 (22.1%)	37 (21.5%)	
Middle cerebral artery	22 (16.8%)	23 (13.4%)	
Anterior cerebral artery	16 (12.2%)	19 (11.0%)	
Posterior cerebral artery	7 (5.3%)	16 (9.3%)	
Vertebral artery	4 (3.1%)	4 (2.3%)	
Surgery	86 (65.5%)	100 (58.1%)	0.183
Aneurysmal size (mm)	9.1 ± 5.7	5.8 ± 3.5	<0.001
Rebleeding	10 (7.6%)	6 (3.5%)	0.110
Acute hydrocephalus	71 (54.2%)	19 (11.0%)	<0.001
Intracerebral hemorrhage	26 (19.9%)	13 (7.6%)	0.002
Intraventricular hemorrhage	52 (39.7%)	20 (11.6%)	<0.001
External ventricular drain	90 (68.7%)	19 (11.0%)	<0.001
Admission time (hours)	4.5 ± 3.4	4.9 ± 3.8	0.342
Seizure	17 (13.0%)	27 (15.7%)	0.505
Systolic arterial pressure (mmHg)	134.2 ± 23.6	128.7 ± 21.2	0.033
Diastolic arterial pressure (mmHg)	81.1 ± 15.1	77.5 ± 13.8	0.031
Mean arterial pressure (mmHg)	98.8 ± 16.1	94.6 ± 15.3	0.020
Plasma C-reactive protein level (mg/L)	7.9 ± 3.0	6.6 ± 2.4	<0.001
plasma D-dimer level (mg/L)	2.2 ± 1.1	1.9 ± 0.9	0.011
Plasma HMGB1 level (ng/mL)	10.6 ± 3.4	7.0 ± 2.9	<0.001

Numerical variables are presented as mean ± standard deviation. Categorical variables are expressed as counts (percentage). Numerical variables were analyzed by Mann–Whitney U-test or unpaired Student *t* test. Categorical variables were analyzed by chi-square test or Fisher exact test. HMGB1, high mobility group box-1; n, number of patients; WFNS, World Federation of Neurological Surgeons.

A receiver operating characteristic curve showed that plasma HMGB1 level on admission statistically significantly predicted cerebrovasospasm of patients (Figure 2C). The predictive value of the HMGB1 concentration was lower than those of WFNS score and modified Fisher score (Table 7). In a combined logistic-regression model, HMGB1 did not statistically significantly improve the area under the curve of WFNS score (*P* = 0.218) or modified Fisher score (*P* = 0.235).

**Table 7:** Receiver operating characteristic curve analysis of factors predicting the cerebrovasospasm among 303 patients

	**HMGB1**	**WFNS score**	**Modified Fisher score**
Criterion	>8.8 ng/mL	>2	>2
Area under curve	0.804	0.879	0.874
95% confidence interval	0.754 to 0.847	0.837 to 0.913	0.831 to 0.909
Sensitivity	74.0	80.9	84.7
95% confidence interval	65.7 to 81.3	73.1 to 87.3	77.4 to 90.4
Specificity	78.5	79.7	76.7
95% confidence interval	71.6 to 84.4	72.9 to 85.4	69.4 to 82.2
+ likelihood ratio	3.44	3.98	3.64
95% confidence interval	3.0 to 3.9	3.6 to 4.4	3.3 to 4.1
- likelihood ratio	0.33	0.24	0.20
95% confidence interval	0.2 to 0.5	0.2 to 0.4	0.1 to 0.3
*P* value	Reference	0.016	0.017

HMGB1, high mobility group box-1; WFNS, World Federation of Neurological Surgeons.

### Poor neurologic function prediction

Ninety patients (29.7%) suffered from poor neurologic outcome (GOS 1–3) in 1 year. Higher plasma HMGB1 level was associated with 1-year poor neurologic outcome, as well as other variables shown in Table 8. When the above variables that were found to be significant in the univariate analysis were introduced into the logistic model, a multivariate analysis selected WFNS score (odds ratio, 4.872; 95% CI 1.945 to 13.760; *P* = 0.004), modified Fisher score (odds ratio, 5.981; 95% CI 2.519 to 15.379; *P* = 0.003) and plasma HMGB1 level (odds ratio, 1.410; 95% CI 1.112 to 1.914; *P* = 0.002) as the independent predictors for 1-year poor neurologic outcome of patients.

**Table 8:** The factors associated with 1-year function outcome

	**GOS 1 to 3**	**GOS 4 to 5**	***P*value**
	**(n = 90)**	**(n = 213)**	
Sex (male/female)	42/48	89/124	0.433
Age (years)	44.7 ± 11.3	43.5 ± 12.8	0.422
WFNS score on admission	3.6 ± 0.7	1.8 ± 0.9	<0.001
Modified Fisher score on admission	3.8 ± 0.8	2.3 ± 0.7	<0.001
Aneurysmal location			0.291
Posterior communication artery	24 (26.7%)	59 (27.7%)	
Internal carotid artery	13 (14.4%)	30 (14.1%)	
Anterior communication artery	17 (18.9%)	49 (23.0%)	
Middle cerebral artery	14 (15.6%)	31 (14.6%)	
Anterior cerebral artery	7 (7.8%)	28 (13.1%)	
Posterior cerebral artery	11 (12.2%)	12 (5.6%)	
Vertebral artery	4 (4.4%)	4 (1.9%)	
Surgery	54 (60.0%)	132 (62.0%)	0.747
Aneurysmal size (mm)	10.4 ± 5.8	5.9 ± 3.7	<0.001
Rebleeding	10 (11.1%)	6 (2.8%)	0.003
Acute hydrocephalus	47 (52.2%)	43 (20.2%)	<0.001
Intracerebral hemorrhage	20 (22.2%)	19 (8.9%)	0.002
Intraventricular hemorrhage	59 (65.6%)	12 (5.6%)	<0.001
External ventricular drain	66 (73.3%)	43 (20.2%)	<0.001
Angiographic vasospasm	71 (78.9%)	60 (28.2%)	<0.001
Computed tomography ischemia	28 (31.1%)	22 (10.3%)	<0.001
Admission time (hours)	4.5 ± 3.5	4.8 ± 3.7	0.577
Seizure	19 (21.1%)	25 (11.7%)	0.085
Plasma C-reactive protein level (mg/L)	8.2 ± 3.2	6.7 ± 2.4	<0.001
plasma D-dimer level (mg/L)	2.4 ± 1.2	1.9 ± 0.9	<0.001
Plasma HMGB1 level (ng/mL)	11.5 ± 3.1	7.3 ± 3.0	<0.001

Numerical variables are presented as mean ± standard deviation. Categorical variables are expressed as counts (percentage). Numerical variables were analyzed by Mann–Whitney U-test or unpaired Student *t* test. Categorical variables were analyzed by chi-square test or Fisher exact test. GOS, Glasgow Outcome Scale; HMGB1, high mobility group box-1; n, number of patients; WFNS, World Federation of Neurological Surgeons.

A receiver operating characteristic curve showed that plasma HMGB1 level on admission predicted 1-year poor neurologic outcome of patients statistically significantly (Figure 2D). The predictive value of the HMGB1 concentration was similar to those of WFNS score and modified Fisher score (Table 9). In a combined logistic-regression model, HMGB1 statistically significantly improved the area under curve of WFNS score (*P* = 0.007) and modified Fisher score (*P* = 0.014).

**Table 9:** Receiver operating characteristic curve analysis of factors predicting 1-year poor functional outcome among 303 patients

	**HMGB1**	**WFNS score**	**Modified Fisher score**
Criterion	>8.9 ng/mL	>2	>2
Area under curve	0.858	0.909	0.902
95% confidence interval	0.813 to 0.895	0.871 to 0.939	0.863 to 0.933
Sensitivity	84.4	96.7	95.6
95% confidence interval	75.3 to 91.2	90.6 to 99.3	89.0 to 98.7
Specificity	77.0	74.7	69.5
95% confidence interval	70.8 to 82.5	68.3 to 80.3	62.8 to 75.6
+ likelihood ratio	3.67	3.81	3.13
95% confidence interval	3.3 to 4.1	3.5 to 4.2	2.8 to 3.5
- likelihood ratio	0.2	0.045	0.064
95% confidence interval	0.1 to 0.3	0.01 to 0.1	0.02 to 0.2
*P* value	Reference	0.104	0.163

HMGB1, high mobility group box-1; WFNS, World Federation of Neurological Surgeons.

## Discussion

This study was conducted to determine if plasma HMGB1 is increased in the circulation of humans with SAH and whether this enhancement correlates with in-hospital mortality, cerebrovasospasm and 1-year poor clinical outcomes in these patients. The admission plasma HMGB1 levels were indeed significantly increased in all patients compared with healthy subjects. Furthermore, an admission plasma HMGB1 level was identified as a reliable and independent marker to predict patients at risk of in-hospital mortality, cerebrovasospasm and 1-year poor clinical outcome. Importantly, the prognostic values of HMGB1 were similar to those of WFNS score and modified Fisher score for in-hospital mortality and 1-year poor clinical outcome, substantiating its potential as a new prognostic biomarker.

HMGB1 is a non-histone DNA binding protein that possesses two HMG boxes that are DNA binding domains [27]. As a chromosomal protein, HMGB1 has been implicated in diverse intracellular functions, including the stabilization of nucleosomal structure and the facilitation of gene transcription [28]. Moreover, some evidence identifies HMGB1 as a cytokine-like mediator of delayed endotoxin lethality and acute lung injury [7,29]. HMGB1 is actively secreted by macrophages and monocytes or released by necrotic cells into the extracellular milieu, where it might be involved in the triggering of inflammation [7,8,29,30]. Recombinant HMGB1 has been found to induce acute inflammation in animal models of lung injury and endotoxemia [7,29], and anti-HMGB1 antibody attenuated endotoxin-induced lethality even when administration of antibody was delayed until after early cytokine response [7,31]. In addition, high serum levels of HMGB1 in patients with sepsis or hemorrhagic shock have been reported to be associated with increased mortality and disease severity [7,32].

HMGB1 is widely expressed in various tissues including the brain [13-15]. Moreover, in the brain, HMGB1 has been reported to be released after cytokine stimulation and to be involved in the inflammatory process after it was administered intracerebroventricularly [16,33]. A recent study demonstrates that HMGB1 is massively released into the extracellular milieu during the acute damaging phase and that extracellular HMGB1 might function as a proinflammatory cytokine, activate microglia, and hence stimulate the release of other cytokines and aggravate brain injury in the postischemic brain [17]. Furthermore, HMGB1^−/−^ necrotic cells have a greatly reduced ability to promote inflammation [8]. Although the mechanism by which HMGB1 exerts its proinflammatory cytokine-like effects in the central nervous system is unknown, previous reports have shown that the activations of several mitogen activated protein kinases and nuclear factor kappa B are involved in the proinflammatory effect of HMGB1 [34-36]. These are downstream molecules of receptors for advanced glycation end product or Toll-like receptor family members, which are important receptors in the HMGB1 signaling process [37-40]. Evidence is rapidly accumulating to suggest that HMGB1 may play an important role in brain injury following stroke. Muhammad and colleagues [41] have demonstrated using a mouse middle cerebral artery occlusion model that HMGB1 engagement of receptors for advanced glycation end product triggers inflammation and infarction leading to ischemic brain injury. Recent studies have also reported elevated HMGB1 levels in the cerebrospinal fluid of SAH patients with poor outcome [24,25]. Moreover, another report also found elevated cytosolic HMGB1 in brain parenchyma of SAH animals [15]. To our knowledge, our present study represents the first report of elevated HMGB1 levels in plasma of SAH patients. In our study, a high WFNS score or modified Fisher score upon admission was strongly correlated with the high plasma HMGB1 level. Our data suggest that plasma HMGB1 level in this early period might reflect the initial hemorrhage insult. Connected with the previous studies, our results suggest HMGB1 may act in concert to promote brain inflammation following SAH.

In this study, a receiver operating characteristic curve showed that plasma HMGB1 level on admission predicted poor functional outcome and mortality after 1 year, and in-hospital mortality of patients obviously. The area under the curve of the HMGB1 concentration was similar to those of WFNS score and modified Fisher score for the prediction of these poor outcomes. In a combined logistic-regression model, HMGB1 improved the area under the curve of WFNS score and modified Fisher score for the prediction of poor functional outcome after 1 year, but not for the prediction of mortality after 1 year or in-hospital mortality. Therefore, the determination of HMGB1 in the plasma of patients on admission provides the ability to distinguish between patients with good and bad outcome. Cerebrovasospasm is regarded as abnormal and prolonged smooth muscle contraction of cerebral arteries; many substances have been involved in the development of cerebral vasospasm following SAH, but the complex mechanism of this arterial narrowing is not yet fully understood [42,43]. The degree of angiographic vasospasm is not always well correlated with the development of neurological deficits in SAH patients, and other influences such as early brain injury due to cortical spreading depression, disruption of the blood–brain barrier, impaired function of the microcirculation, inflammation, and apoptotic cell death may contribute to SAH-induced pathologies [44-48]. This study found that plasma HMGB1 level was an independent predictor for cerebrovasospasm of patients . However, significantly lower accuracy for the prediction of cerebrovasospasm was found for plasma HMGB1 level compared with other clinical grade such as WFNS and modified Fisher score. Hence, plasma levels of HMGB1 on admission are not recommended for the prediction of cerebrovasospasm after SAH. Overall, plasma HMGB1 level has low accuracy for the prediction of cerebrovasospasm and high accuracy for the prediction of 1-year mortality or in-hospital mortality or 1-year poor functional outcome. However, HMGB1 determination does not add to the accuracy of prediction of the clinical outcomes.

In addition, location of the subarachnoid blood can be assessed in the following four anatomical locations: convexity, sylvian fissure, basal cistern, and interhemisphere. However, most of the subarachnoid blood resides in multiple anatomical locations leading to difficulty of classification. Hence, whether HMGB1 levels differ according to SAH location warrants further investigation; maybe this is a limitation in this study. Actually, subarachnoid blood is impossible to gather in just an anatomical location. We need to mention that, in this study, ischemic or hemorrhage stroke referred to stroke in the patients’ history. Patients with previous neurological diseases including ischemic or hemorrhagic stroke, or the use of antiplatelet or anticoagulant medication, were excluded because these patients were complicated with vascular risk factors including diabetes mellitus and/or hypertension that were associated with high HMGB1 level [49-52] and became the confounding variables.

## Conclusions

In this study, plasma HMGB1 level is a useful, complementary tool to predict functional outcome and mortality after aneurysmal SAH. However, HMGB1 determination does not add to the accuracy of prediction of the clinical outcome.

## Abbreviations

CI, confidence interval; CT, computerized tomography; ELISA, enzyme-linked immunosorbent assay; GCS, Glasgow Coma Scale; GOS, Glasgow outcome scale; HMGB1, high mobility group box-1; SAH, subarachnoid hemorrhage; WFNS, World Federation of Neurological Surgeons.

## Competing interests

The authors declare that they have no competing interests.

## Authors’ contributions

XDZ and JSC contributed to the design of the study and drafted the manuscript and participated in the laboratory work. JSC, FZ and QCL enrolled the patients. GC and JMZ contributed to data analysis and interpretation of the results. All authors read and approved the final manuscript.
